# The potential cost-effectiveness of HPV vaccination among girls in Mongolia

**DOI:** 10.1016/j.jvacx.2022.100161

**Published:** 2022-04-08

**Authors:** Munkh-Erdene Luvsan, Elisabeth Vodicka, Uranbolor Jugder, Undarmaa Tudev, Andy Clark, Devin Groman, Dashpagam Otgonbayar, Sodbayar Demberelsuren, D. Scott LaMongtagne, Clint Pecenka

**Affiliations:** a Department of Health Policy, School of Public Health, Mongolian National University of Medical Sciences, Rm. 334. Sukhbaatar District, S.Zorig Street, Ulaanbaatar, Mongolia; b PATH, Seattle, WA, USA; c Department of Cancer Registry Surveillance, Early Detection, National Cancer Center, Nam-Yan Ju Street, Bayanzurkh District, Ulaanbaatar 13370, Mongolia; d London School of Hygiene and Tropical Medicine, Department of Health Services Research and Policy, London, United Kingdom; e Department of Immunization, National Center for Communicable Disease, Ministry of Health, Nam-Yan Ju Street 32/1, Bayanzurkh District, Ulaanbaatar 13335, Mongolia; f World Health Organization Representative Office Mongolia, Government Building VIII, Olympic Street 2, Sukhbaatar District, Ulaanbaatar 14210, Mongolia

**Keywords:** HPV, Vaccination, Cost-effectiveness, DALY, ICER, Mongolia

## Abstract

**Introduction:**

Cervical cancer is a leading cause of cancer among women in Mongolia with an age-standardized incidence rate of 23.5 per 100,000. HPV vaccination has not been introduced nationally and Gavi co-financing support is not available in Mongolia. Extended Gavi pricing for HPV vaccine may be available from vaccine manufacturers for a number of years. To inform introduction decision-making, we evaluated the potential cost-effectiveness of HPV vaccination among girls and young women in Mongolia.

**Methods:**

We used UNIVAC (version 1.4), a static decision model, to evaluate the health and economic outcomes of single-cohort vaccination among females from the government perspective compared to no vaccination. We modeled vaccine introduction over 10 birth cohorts starting in 2022 comparing quadrivalent or bivalent vaccine selection and vaccine pricing variations. We used locally-specific data for cancer incidence, mortality, treatment and costs. Model outcomes included cancer cases, hospitalizations, deaths, disability-adjusted life years (DALY), and costs presented in 2018 USD. Incremental costs and health outcomes were discounted at 3% and aggregated into an Incremental Cost-Effectiveness Ratio (ICER).

**Results:**

The base-case scenario of HPV vaccination among 9 year-old girls was projected to avert 5,692 cervical cancer cases, 3,240 deaths, and 11,886 DALYs and incur $2.4–3.1M more costs compared to no vaccination. At prices of ($4.50-$4.60/dose), we estimated an ICER of $166-$265/DALY averted among 9-year-olds. When price per dose was increased to reported mean vaccine purchase price for non-Gavi LMICs ($14.17/dose), the ICER ranged from $556–820/DALY averted.

**Conclusion:**

HPV vaccination among girls is highly likely to be a cost-effective investment in Mongolia compared to no vaccination with projected ICERs less than 20% of the 2018 GDP per capita of $3,735.

## Introduction

Human papillomavirus (HPV) is a globally pervasive virus that can lead to the development of cervical cancer. In 2018, the International Agency for Research on Cancer (IARC) estimated approximately 570,000 new cases of cervical cancer each year with over 85% of the burden occurring in low- and middle-income countries (LMICs) [1]. In Mongolia, cervical cancer is the second leading cause of cancer among women with an age-standardized incidence rate of 23.5 per 100,000 and an age-standardized mortality rate of 10.2 per 100,000 [2]; it is the most common cancer among women of reproductive age (15–44 years). Each year, about 370 new cervical cancer cases are diagnosed and 150 cervical cancer deaths occur among women in the country (estimates for 2018) [3]. Of those who develop cancer, estimates from 2015 national cancer registry data indicate that over half of patients are diagnosed late stage with a 5-year survival rate of 44% [4].

While early detection of disease through screening and treatment of pre-cancerous lesions can halt disease progression and reduce cancer-related morbidity and mortality, approximately 30% of women of reproductive age have ever received screening in Mongolia [3]. This makes primary prevention through safe and efficacious vaccines critical to cervical cancer disease prevention. Current vaccines on the market have been shown to protect against HPV types that are responsible for over 70% of cervical cancers and pre-cancerous cervical lesions, with some vaccines also providing cross-protection of non-vaccine targeted HPV genotypes [5]. Given the promising available strategies to prevent, detect and treat cervical cancer, in 2020 the World Health Organization has launched a global strategy for the elimination of cervical cancer through scale-up of effective vaccination, screening and treatment programs [6]. As part of this global effort, the Mongolian Ministry of Health is making strides toward expanding primary and secondary prevention of cervical cancer and treatment [7].

While HPV vaccination is likely to be a cost-effective public health investment in both low-income and high-income countries, most national introductions have occurred in high- or upper-middle-income countries. In LMICs, HPV vaccine implementation has primarily occurred via small-scale pilots or demonstration projects with significant financial and programmatic challenges identified as barriers to national scale-up [8]. In 2012, the Mongolian Ministry of Health and the Millennium Challenge Corporation (Washington DC, USA) organized a pilot HPV vaccine introduction program for schoolgirls aged 11 to 15 years. However, concerns about the side effects of the HPV vaccines led to the interruption of the pilot program in the same year [9]. In Mongolia, a nationally organized HPV vaccination program has not been initiated and Gavi co-financing support is not available, although extended Gavi pricing for HPV vaccine may be available through pricing commitments from vaccine manufacturers for a number of years.

More information is needed to support countries in deciding whether to incorporate HPV vaccination into their national immunization programs within the context of funding constraints and competing health and investment priorities. Estimating the potential health and economic impact through cost-effectiveness analysis can provide local decision-makers with evidence to identify potentially high-value vaccines and inform decisions regarding national vaccine introduction. Through this study, we conducted an HPV cost-effectiveness analysis in Mongolia to evaluate the potential health and economic impact of HPV vaccination among girls across a variety of potential introduction strategies.

## Materials and methods

### Model

We used UNIVAC (version 1.4), a static proportional outcomes model, to estimate the incremental lifetime costs and health outcomes associated with vaccination using the Gardasil® quadrivalent (Merck) or Cervarix™ bivalent (GSK) vaccines through several potential introduction scenarios compared to no vaccination. Results were modeled over the lifetime horizon from the government and societal perspectives [10]. The UNIVAC model has previously been used to estimate the potential health and economic impact of rotavirus vaccine introduction in Mongolia [11], and has been used to evaluate HPV vaccine strategies elsewhere [12], [13]. For HPV, model inputs include cervical cancer incidence and mortality by stage, vaccine efficacy, coverage and costs (commodities and program delivery costs), as well as health care costs associated with cervical cancer treatment by stage. The cost-effectiveness analysis combined incremental costs and outcomes to assess value through the calculation of an Incremental Cost-Effectiveness Ratio (ICER), reported as the cost per disability-adjusted life year (DALY) averted. Other outcomes include cervical cancer cases by stage, hospitalizations, deaths, healthcare costs and vaccine program costs.

Costs and outcomes were discounted at 3%. Discounting reflects the notion that the value of costs and health outcomes that occur in the future are less than the value of costs and health outcomes that we experience presently. Therefore, the discount rate takes this preference into consideration and offsets the value of future effects. All monetary units are presented in constant 2018 United States dollars (USD).

### Scenarios evaluated

In close collaboration with local stakeholders at the Expanded Programme on Immunization (EPI) of the National Center for Communicable Diseases (NCCD), National Cancer Center of Mongolia (NCC), Ministry of Health (MOH), and the World Health Organization (WHO), we identified a realistic base case (most likely) introduction scenario, developed potential alternative introduction scenarios of interest, and then iteratively estimated, reviewed, and finalized all parameters in the model. For this analysis, the base case scenario was developed as the most likely scenario to include vaccinating 9-year-old girls using the quadrivalent vaccine with a flat rate of $4.50 per dose, under the assumption that Gavi pricing at the time of the analysis would be extended to Mongolia under commitments from vaccine manufacturers [14]. We assumed nationwide introduction beginning in 2022 over 10 consecutive birth cohorts with no catch-up campaign. Thus, vaccination was modeled to occur over the period 2022–2031 with costs and health outcomes tracked over girls’ lifetime horizons. Expected delivery strategies for girls included a 2-dose schedule via schools (70%), outreach (10%), and health facilities (20%).

We also examined the potential cost-effectiveness of HPV vaccine introduction across a variety of scenarios using a combination of input parameters that varied vaccine selection and vaccine pricing. These scenarios were chosen based on stakeholder interest and to inform decision-making.

### Disease burden

Age-stratified data from 2018 on cancer incidence, treatment by stage, and mortality were obtained from the NCC and used to calculate age-specific cumulative incidence and mortality rates per 100,000 women (Table 1). These data reflected more current information than the underlying NCC data currently used for Mongolia in the GLOBOCAN registry (2018), which were calculated using disease event rates from 2012 over the 2018 population. Our model did not include data on cervical precancer or the impacts of screening and treatment for cervical intraepithelial neoplasia.

**Table 1:** Annual age-specific cervical cancer burden per 100,000 (by stage) in 2018.

**Age**	**Total ASRI**	**Local ASRI**	**Regional ASRI**	**Distant ASRI**	**Mortality**	**Source**
*Age-specific rates per 100,000*†						
<24	0	0	0	0	0	Estimated from National Cancer Center of Mongolia 2018 registry data. [4]
25–29	4.9	2.1	2.8	0.0	2.1	
30–34	15.9	9.7	4.8	1.4	0.7	
35–39	33.9	18.6	14.4	0.8	4.2	
40–44	48.1	21.8	20.8	5.6	9.3	
45–49	51.5	19.1	25.3	7.2	17.5	
50–54	68.6	23.3	39.5	5.8	19.8	
55–59	69.4	17.4	45.1	6.9	29.2	
60–64	70.2	23.4	31.9	14.9	48.9	
65–69	86.2	20.7	58.6	6.9	62.1	
70–74	63.2	13.2	39.5	10.5	52.6	
75+	77.8	14.8	44.4	18.5	77.8	

† We used 2018 incident case estimates for each disease event to calculate age-specific mortality and cumulative incidence rates. The denominator was the age-specific female population for 2018 based on World Bank data. Mortality and incident case data were provided by NCC. We collapsed ages 0–24 years old and 75+ years old and applied the same event rates to all individuals in those respective categories. 25–74-year-old age group estimates were based on the specific estimates for each 5-year age category. Case rates based on TNM staging were converted to local (37%), regional (52%) and distant (11%) invasive cancer rates in consultation with local experts and the NCC. The cancer registry data did not distinguish between TNM stage 2a (local invasive cancer) and TNM stage 2b (regional invasive cancer) cases. Thus, we assumed that 50% of Stage 2 cases were 2a (local) and 50% were 2b (regional).

Cervical cancer staging in Mongolia is typically conducted using TNM staging system (evaluation of the primary tumor, lymph nodes and metastasis), while the UNIVAC model categorizes invasive cervical cancer as local, regional or distant. Therefore, local clinical experts were consulted to categorize typical TNM staging to local (TNM stages T1-T2a), regional (T2b-T3) and distant invasive (T4) cancer for purposes of the model, recognizing that each cancer case is nuanced and may not map perfectly to a 3-stage system. Based on incident case data from the NCC registry and in consultation with local experts, we estimated that 37% of cases were locally invasive, 52% were regionally invasive and 11% distant invasive.

### Vaccine coverage and efficacy

We estimated a vaccine coverage rate of 93% (74–100%) based on data from the EPI (Table 2). This coverage was assumed to be the same for both doses. Vaccines protect at different levels against each HPV genotype [5]. Therefore, we also calculated a weighted efficacy based on the estimated genotype prevalence in Mongolia [9] and type-specific efficacy [5] to assess the impact of cross-protection against non-vaccine HPV genotypes (Supplementary Appendix Table 1). Type specific efficacy was multiplied by the share of burden for the respective type and then summed over the cervical cancer associated types relevant for each product. Using the quadrivalent product as an example, for types 16 and 18, vaccine efficacy was 94% (92–97%) for HPV- naïve girls from the average vaccine efficacy in HPV-naïve study populations from FUTURE and PATRICIA trials [14]. This value was multiplied by the share of cervical cancer associated disease burden attributable to types 16 and 18 (64.3%) [9]. We also accounted for 70% efficacy for type 31 accounting for 7.1% of the cervical cancer associated disease burden in Mongolia [5], [9]. This yields an overall quadrivalent efficacy of 65.6% against cervical cancer associated HPV types. The same method was applied to the bivalent product. Note that the quadrivalent vaccine also protects against HPV 6 and 11 which cause genital warts. These benefits and other non-cervical cancer associated disease are excluded in this analysis.

**Table 2:** Key model parameters for evaluating the cost-effectiveness of HPV vaccine.

**Parameter**	**Base case (range)**	**Source/s**
**Estimated prevalence of high-risk HPV genotypes with targeted or high vaccine cross-protection (% among all cervical cancers)**		
16/18	64.3%	[9]
31	7.1%	[9]
33	14.3%	[9]
45	Not identified	[9]

**Disability weights for cervical cancer by stage**		
Local	28.8% (19.3–39.9%)	[16] Diagnosis and primary therapy phase of cervical cancer
Regional	45.1% (30.7–60.0%)	[16] Metastatic phase of cervical cancer
Distant	54% (37.7–68.7%)	[16] Terminal phase of cervical cancer

**Estimated mean duration of cervical cancer illness by stage (years)**^a^		
Local	15 (7.5–22.5)	Assumption based on SurvCan data from India [17] and expert opinion
Regional	7.5 (3.75–11.25)	Assumption based on SurvCan data from India [17] and expert opinion
Distant	2.5 (1.25–3.75)	Assumption based on SurvCan data from India [17] and expert opinion

**Vaccine coverage**		
Dose 1	93% (74–100%)	EPI and 2018 WHO Technical Report (unpublished)
Dose 2	93% (74–100%)	EPI and 2018 WHO Technical Report (unpublished)

**Vaccine efficacy 2 weeks after vaccination**		
2-dose efficacy among girls		[5], [9] Pooled odds ratio estimates from PATRICIA & FUTURE trials weighted by vaccine type coverage and genotype prevalence in Mongolia
Gardasil®	65.6% (61.2–68.5%)	
Cervarix™	78.8% (71.3–82.8%)	

**HPV vaccination program costs**		
International handing (% of vaccine price)	4%	Assumption
International delivery (% of vaccine price)	15%	Assumption
HPV vaccine wastage	5%	Derived from 2018 Technical Report and EPI
Syringe and safety box price per dose	$0.05	Derived from 2018 Technical Report and EPI
HPV vaccine price per dose	Girls’ program	*Girls’ program* Base case: Gavi extended pricing for quadrivalent vaccine [14]; High range: Average HPV price per dose reported for self-procurement among non-Gavi LMICs [15]
Gardasil®	$4.50 ($0–14.17)	
Cervarix™	$4.60 ($0–14.17)	
Vaccine delivery cost per dose by year	Girls’ program	Base case estimates calculated based on data from EPI and 2018 Technical Report for annual birth cohort population Range: ±50% base case
2022 (year of introduction)	$14.25 ($7.13–21.38)	
2023	$7.07 ($3.54–10.61)	
2024	$6.95 ($3.48–10.43)	
2025	$6.89 ($3.44–10.33)	
2026	$6.87 ($3.44–10.31)	
2027	$6.85 ($3.43–10.28)	
2028	$6.84 ($3.42–10.26)	
2029	$6.83 ($3.41–10.24)	
2030	$6.82 ($3.41–10.22)	
2031	$6.81 ($3.4–10.21)	

**Cervical cancer treatment costs**		
Local	$2,355 ($1,178–3,533) (Government);$2,413 ($1,207–3,620) (Societal)	Calculated based on 2018 Technical Report and NCC expert opinion. Range: ±50%
Regional	$4,310 ($2,155–6,465) (Government);$4,368 ($2,184–6,552)(Societal)	Calculated based on 2018 Technical Report and NCC expert opinion. Range: ±50%
Distant	$2,882 ($1,441–4,323) (Government);$2,946 ($1,473–4,491) (Societal)	Calculated based on 2018 Technical Report and NCC expert opinion. Range: ±50%

a Due to limited survival data, base case average duration of illness was estimated using SurvCan data from India [18] using the Declining Exponential Approximation of Life Expectancy method to convert five-year survival rates into average duration of life expressed in years [17]. Low and high range estimates were calculated ±20% of base case estimate.

### Vaccine delivery cost and price

We estimated the year-over-year health systems costs to deliver HPV vaccine to girls based on existing EPI and local cost data. Delivery costs included introduction costs for social mobilization, training, monitoring and evaluation, advocacy, communications, and program management, as well as ongoing staff time, transportation and other costs required to provide vaccination through schools, facilities and outreach. We assumed no cost to the household for vaccine delivery.

For vaccine pricing, we applied a Gavi price per dose at the time of the analysis of $4.50 for the Gardasil® quadrivalent vaccine and $4.60 for Cervarix™ bivalent vaccine. Although Mongolia is no longer Gavi-eligible, Merck extended Gavi prices for GARDASIL® quadrivalent vaccine through 2025 to countries which are transitioning or have fully transitioned from Gavi support [14]. Among others, this extension applied to Gavi countries with Gross National Income (GNI) per capita of over US$3,200 that entered accelerated transition phase before the HPV vaccine support application window and Mongolia was identified as a potentially eligible country [14]. However, ability to access this pricing will need to be confirmed. As an alternative, we also explored the results assuming a price increase in 2026 (expected sunset of extended Gavi pricing) or higher price of vaccine from the very start applying the mean of reported vaccine pricing per dose for non-Gavi LMICs ($14.17 per dose, range: $13.69–14.87) [15].

### Costs of cervical cancer treatment

Treatment costs of invasive cancer were based on previously collected cost data provided by the WHO Country Office and expert opinion. The government perspective included direct medical costs (supplies/equipment, staffing, medications, etc.) and direct non-medical costs (overhead). Societal perspective included all direct costs, plus indirect costs of a woman’s time spent seeking staging, diagnosis and treatment estimated by duration of procedures (outpatient services) or number of bed days (inpatient services). For the opportunity cost of a woman’s time, we used the monthly minimum wage in Mongolia (320,000 MNT or $120 USD) multiplied by the number of days required for treatment. Locally invasive cancer was expected to be treated primarily by hysterectomy alone, with a proportion of women receiving radiotherapy, chemotherapy, and hysterectomy either concomitantly or alone. Regional invasive cancer was expected to be treated primarily with a combination of hysterectomy plus radiotherapy, with some women also receiving chemotherapy. Distant invasive cancer was expected to be treated primarily with radiotherapy and/or palliation. We assumed that the woman would miss an entire day of work for each day of treatment. Of note, societal level costs do not include expected costs associated with transportation, meals or accommodations, caregiver spillover costs, or non-health related costs due to lack of data on these factors.

### Sensitivity analysis

Univariate and probabilistic sensitivity analyses were conducted to evaluate the impact of parameter uncertainty on model outcomes, respectively, by varying model parameters individually or jointly over their respective ranges. Probabilistic sensitivity analyses were conducted over 5,000 Monte Carlo simulations to obtain 95% credible ranges for estimates. Where possible, ranges were defined as measures of variance provided by the original sources (e.g., standard errors for pooled odds ratios) or observed low/high ranges (e.g., minimum/maximum regional vaccine coverage rates). In absence of data, we estimated lower and upper ranges as ±50% of the base case estimate unless otherwise indicated. Univariate results are presented as tornado diagrams and probabilistic results are presented via a cost-effectiveness acceptability curve.

## Results

**Fig. 1:**
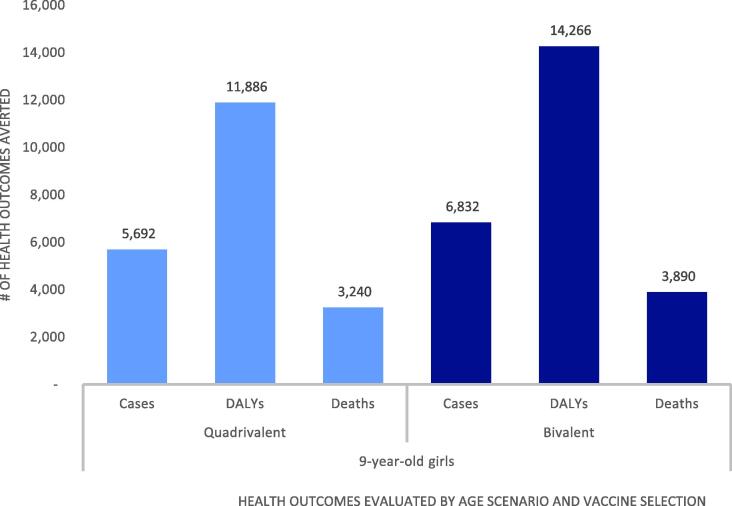
Cervical cancer cases, DALYs and deaths averted with vaccination over 10 birth cohorts compared to no vaccination by scenario.

National introduction of HPV vaccine was projected to improve health outcomes associated with cervical cancer compared to no vaccination (Fig. 1). Under the base case scenario of quadrivalent vaccine targeting 9-year-old girls, we projected that vaccination would avert 1,751 cases of locally invasive cancer, 3,148 cases of regional cancer, 793 cases of distant cancer, 3,240 cervical cancer deaths, and 11,886 DALYs compared to no vaccination over 10 birth cohorts. When the bivalent vaccine was considered for administration among the same age group, we projected that vaccination over 10 birth cohorts would prevent approximately 20% more cancer cases, DALYs and deaths than the quadrivalent vaccine (2,101 local, 3,778 regional, 952 distant cancer cases; 14,266 DALYs; 3,890 deaths) compared to no vaccination due to bivalent cross-protection against non-vaccine targeted HPV genotypes present in the Mongolian population. While use of the bivalent vaccine was expected to avert more health outcomes than the quadrivalent vaccine, these results should be interpreted recognizing not all HPV vaccine benefits have been considered in this analysis. In particular, the quadrivalent vaccine protects against HPV 6 and 11 which cause genital warts. These benefits are excluded in this analysis.

Table 3 presents the projected vaccine program costs, cervical cancer treatment costs with and without vaccination, and total incremental costs compared to no vaccination by scenario (vaccine selection and pricing assumptions) from both the government and societal perspectives. Expected government costs to implement a national vaccine program over 10 birth cohorts were estimated to range from $7.4 to $14.0M, depending on the strategy. When downstream costs of cervical cancer treatment averted were considered from the government perspective, vaccination of 9-year-old girls with fixed vaccine pricing at extended Gavi rates ($4.50 for quadrivalent and $4.60 for bivalent) year-over-year for 10 birth cohorts was projected to cost $2.4M and $3.1M more than no vaccination with the quadrivalent and bivalent vaccines, respectively. This includes $7.4M for vaccine program costs offset by cervical cancer treatment costs averted of $4.2–5.1M over the lifetime horizon.

**Table 3:** Cost outcomes by scenario for vaccination over 10 birth cohorts evaluated from government and societal perspectives.

**Target Age**	**Vaccine Selection**	**Vaccine Price Scenario Evaluated (price per dose)**	**Cost per DALY averted**		**Total incremental costs**†		**Vaccine Program Costs**	**Cervical Cancer Treatment Costs**		
			**Gov.**	**Soc.**	**Gov.**	**Soc.**		**Without** **vaccine**	**With vaccine**	**Costs averted with vaccine**
9-year-old girls	HPV4	$4.50 annually	$265	$259	$3,146,680	$3,074,336	$7,378,136	$6,933,639 (G);$7,052,181 (S)	$2,702,183 (G);$2,748,381 (S)	$4,231,456 (G);$4,303,800 (S)
		$4.50 annually until 2026; $14.17 thereafter	$569	$563	$6,760,331	$6,687,987	$10,991,787			
		$14.17 annually	$820	$814	$9,749,038	$9,676,694	$13,980,494			
	HPV2	$4.60 annually	$166	$160	$2,367,468	$2,280,635	$7,446,413		$1,854,694 (G);$1,886,403 (S)	$5,078,945 (G);$5,165,778 (S)
		$4.60 annually until 2026; $14.17 thereafter	$417	$411	$5,943,749	$5,856,916	$11,022,694			
		$14.17 annually	$624	$618	$8,901,549	$8,814,716	$13,980,494			
		$14.17 annually, increase 5% per year	$2,138	$2,131	$6,718,486	$6,699,194	$7,847,477			

HPV4 = Quadrivalent vaccine; HPV2 = Bivalent vaccine; G = government perspective; S = societal perspective.

† Total incremental costs = Vaccine program costs less cervical cancer treatment costs averted by vaccination.

Incremental costs and DALYs were combined to calculate a cost per DALY averted for each scenario (Table 3). At Gavi pricing ($4.50 and $4.60/dose annually for quadrivalent and bivalent vaccines, respectively), we estimated an ICER of $166 per DALY averted with the bivalent vaccine and $265 per DALY averted among 9-year-olds with the quadrivalent vaccine from the government perspective. When price per dose was increased to the WHO Vaccine Product, Price and Procurement (V3P) mean price for non-Gavi LMICs ($14.17/dose annually) starting in year 2026 or for all years from the start of vaccination, the ICER ranged from $417-$820 per DALY averted when targeting 9-year-old girls from the government perspective. Inclusion of societal-level costs resulted in slightly more favorable ICERs for all scenarios due to the additional cervical cancer treatment costs averted when productivity losses were considered.

### Sensitivity analyses

**Fig. 2:**
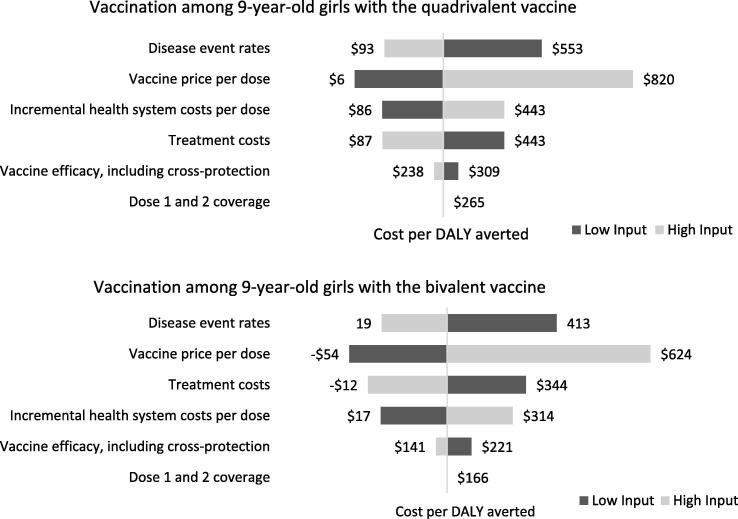
One-way sensitivity analyses results over 10 birth cohorts from the government perspective assuming no year-over-year change in vaccine price.

**Fig. 3:**
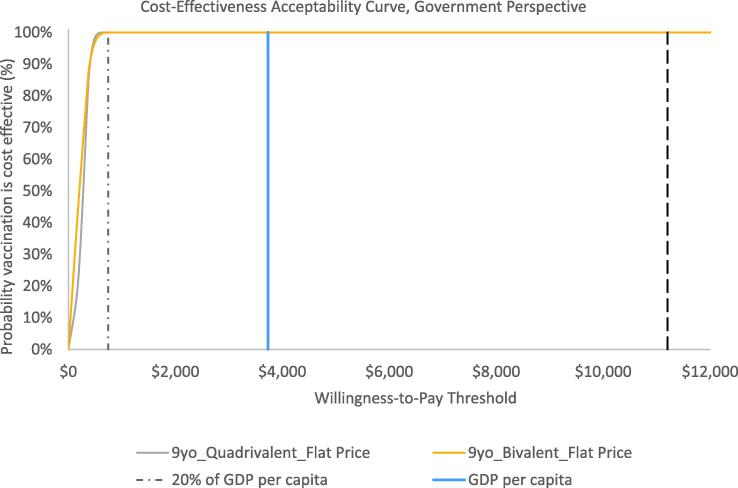
Cost-effectiveness acceptability curves demonstrating probability that vaccination is cost-effective at various willingness-to-pay thresholds by scenario from the government perspective.

Results from the one-way sensitivity analyses suggest that the vaccine price per dose, disease event rates, and costs of vaccine delivery and cervical cancer treatment were the most influential parameters on outcomes (Fig. 2). In general, the probabilistic sensitivity analyses suggest that results are robust to uncertainty. Fig. 3 presents findings as a cost-effectiveness acceptability curve demonstrating the likelihood that vaccination would be cost-effective across a wide-range of willingness-to-pay thresholds from the government perspective. As shown in the CEAC, vaccination among girls assuming flat pricing was projected to be cost-effective in 100% of 5,000 Monte Carlo simulations at WTP thresholds of at least $747 per DALY averted from either perspective. This corresponds to 20% of the 2018 GDP per capita.

## Discussion

HPV vaccination among girls is likely to be a cost-effective investment in Mongolia compared to no vaccination with projected deterministic ICERs as low as 4% of the GDP per capita of $3,735 across scenarios. Based on currently available data on local HPV genotyping among cervical cancer cases in Mongolia [9], the bivalent vaccine may generate better value than the quadrivalent due to relevant cross-protection depending on vaccine price and other factors. A local prevalence study indicated that HPV types 16 and 18, the two genotypes specifically targeted by both the bivalent and quadrivalent vaccines, are prevalent in 64.3% of cervical cancer cases in Mongolia [9]. The bivalent vaccine has been shown to provide high-levels of cross-protection against high-risk HPV types 31 and 33, which were prevalent in 7.1% and 14.3% of cervical cancer cases locally [5], [9]. Inclusion of cross-protection and underlying HPV genotyping likely contributed to our findings that scenarios with the bivalent vaccine were more favorable than scenarios with the quadrivalent vaccine when compared to no vaccination, despite the higher vaccine Gavi price point for the bivalent vaccine ($4.60 per dose vs. $4.50 per dose) at the time of the analysis. As decision-makers consider whether to introduce HPV vaccine at a national level, the level of cross-protection provided by different vaccine products may warrant consideration in addition to the evolving evidence on local HPV genotype prevalence and vaccine prices available to Mongolia at the time of introduction. However, it is to be noted that the quadrivalent benefits of preventing diseases (genital warts) associated with HPV types 6 and 11 were not included in this analysis focused on cervical cancer associated HPV vaccine types. Including these benefits would increase the value of the quadrivalent vaccine.

Importantly, the choice of vaccine should also include discussions of vaccine availability and affordability. The nonavalent vaccine was not evaluated due to its high price point, as it was determined to be unaffordable by the MOH. For scenarios with the bivalent and quadrivalent vaccine, costs of a national vaccine program were estimated to range from $6.7 to $14.0M, depending on strategy, over 10 birth cohorts. Recently published analyses suggest that these estimated HPV vaccine program costs are comparable to those estimated for national programs under consideration or recently introduced in Mongolia for routine immunization against other antigens. For example, estimated costs for a national rotavirus vaccination program over 10 birth cohorts were projected to range from $6 to $11M (2017 USD) depending on vaccine choice [11]. Similarly, estimated costs for national vaccination against pneumococcal using the 13-valent pneumococcal conjugate vaccine (PCV-13) have been projected at $920,000 in the first year and $820,000 in subsequent years, or approximately $8.3M (2016 USD) over 10 years [18].

**Fig. 4:**
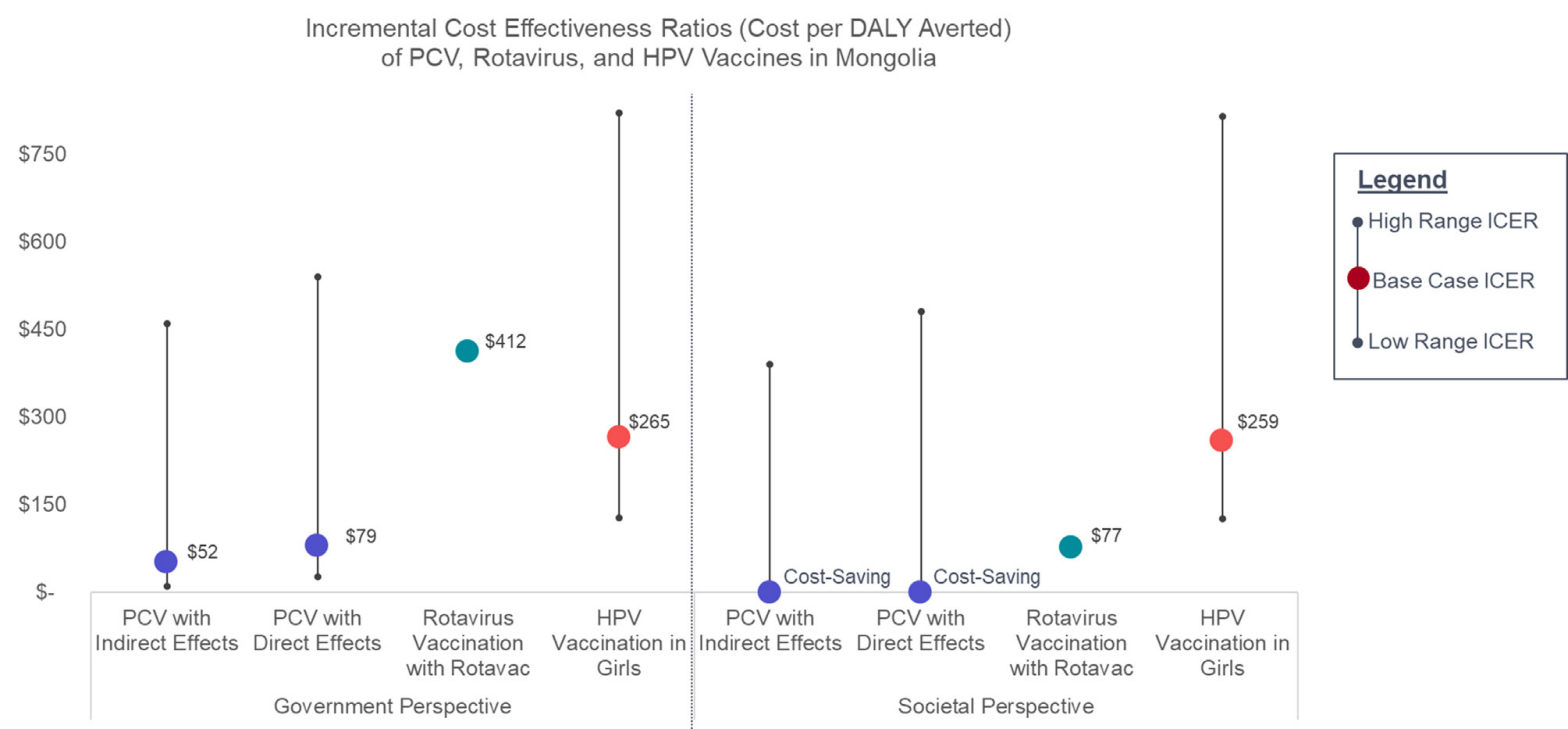
Comparison of HPV Vaccine cost-effectiveness results compared to other vaccine economic evidence in Mongolia.

While an explicit cost-effectiveness threshold has not been established in Mongolia, our probabilistic sensitivity analysis suggests that national HPV immunization of 9-year-old girls, with deterministic ICERs ranging from $166 to $820 across scenarios from the government perspective, is highly likely to be cost-effective at most WTP thresholds and 100% of the time at a threshold of at least $747 per DALY averted. HPV vaccine CEA results can also be considered alongside economic evidence for other vaccines previously identified as good value for money. PCV-13 was introduced into Mongolia’s routine immunization program through a phased approach starting in 2016 [19], and rotavirus vaccination is currently under consideration for introduction. A cost-effectiveness analysis of PCV vaccination among infants in Mongolia suggested that PCV was a high-value vaccine with a cost per DALY averted ranging from $52 to $540 (2014 USD) from the health system perspective and cost-saving to $480 per DALY averted from the societal perspective, depending on pricing assumptions and inclusion or exclusion of indirect herd effects [18]. Similarly, a CEA of rotavirus vaccination conducted using the UNIVAC model resulted in a cost per DALY ranging from $412 to $1,050 from the government perspective and $77 to $715 from the societal perspective (2017 USD), depending on product choice [11]. Comparing the economic evidence from this analysis of HPV vaccination to that of PCV and rotavirus vaccination, HPV vaccination among girls is expected to yield similarly good value for money (Fig. 4).

Finally, cost-effectiveness ratios can be compared to empirically estimated cost-effectiveness thresholds for Mongolia, which suggests that local investments may yield good value for money at a cost per DALY averted of less than $543-$2,805 (2013 USD) [19]. Compared to the 2013 GDP per capita in Mongolia of $4,366, this represents a cost per DALY averted of less than 12–48% of GDP per capita. Our probabilistic sensitivity analysis results for HPV vaccination among girls suggests a cost per DALY averted below 20% of 2018 GDP per capita.

We note several limitations to our analysis. First, the model does not incorporate potential benefits from “herd effects”, however, some studies suggest that the vaccine may confer protection to unvaccinated populations [20], [21], [22]. Thus our results are conservative. Importantly, the model also does not incorporate the costs or health effects associated with cervical cancer screening, detection and treatment of cervical intraepithelial neoplasia. In Mongolia, approximately 30% of women 15–49 have received screening at least once per lifetime [3]. The presence of an active national screening may interact with the value of vaccination and impact the potential cost-effectiveness results; however, economic models of comprehensive cervical cancer prevention programs that include both vaccination and screening and treatment for pre-cancerous lesions have shown that vaccination of girls is generally still highly cost-effective and can yield greater health impact when combined with screening for adult women [23], [24], [25]. This analysis also focuses on HPV vaccine types associated with cervical cancer so the benefits of preventing HPV-associated cancers at other anatomical sites as well as genital warts are excluded. In addition, we use a consistent value for incremental delivery costs that does not vary with coverage. This has the potential to mute the effect of coverage in our one-way sensitivity analysis because it does not account for the effect of fixed costs being spread over a different number of doses delivered. Finally, the societal perspective likely underestimates the household-level costs associated with cervical cancer treatment. It also excludes the costs incurred by households to receive vaccination.

Despite the limitations, our model incorporates locally-specific data on costs, programming, disease burden, and HPV genotyping, which provides decision-makers with an important understanding of the potential health and economic impacts that could be achieved by introducing HPV vaccination at a national scale across scenarios informed by and relevant to key stakeholders in Mongolia. We found that introduction of HPV vaccination into the national EPI would be cost-effective compared to no vaccination. Importantly, economic evidence needs to be considered alongside budget impact, affordability, feasibility, equity, alternative interventions, and other local considerations.

## Source of funding

This work was supported, in whole or in part, by the Bill & Melinda Gates Foundation [OPP1147721].

Under the grant conditions of the Foundation, a Creative Commons Attribution 4.0 Generic License has already been assigned to the Author Accepted Manuscript version that might arise from this submission.

## Declaration of Competing Interest

The authors declare that they have no known competing financial interests or personal relationships that could have appeared to influence the work reported in this paper.

## References

[1] International Agency for Research on Cancer Fact Sheet, Mongolia. Glob Cancer Obs World Heal Organ; 2019. http://gco.iarc.fr/today/data/factsheets/populations/496-mongolia-fact-sheets.pdf (accessed March 12, 2019).

[2] Population Fact Sheet: Mongolia. International Agency for Research on Cancer, The Global Cancer Observatory, World Health Organization. January 2019; n.d.. Available online at: http://gco.iarc.fr/today/data/factsheets/populations/496-mongolia-fact-sheets.pdf.

[3] Bruni L, Albero G, Serrano B, Mena M, Gómez D, Muñoz J, Bosch FX, de Sanjosé S. ICO/IARC Information Centre on HPV and Cancer (HPV Information Centre). Human Papillomavirus and Related Diseases in Mongolia. Summary Report 17 June 2019. [Accessed 7 Aug 202. n.d.].

[4] National Center for Cancer of Mongolia. National Registry Data for Cancer; n.d.

[5] Malagón T., Drolet M., Boily M.-C., Franco E.L., Jit M., Brisson J. (2012). Cross-protective efficacy of two human papillomavirus vaccines: A systematic review and meta-analysis. Lancet Infect Dis.

[6] Global strategy to accelerate the elimination of cervical cancer as a public health problem. Geneva: World Health Organization; 2020. Licence: CC BY-NC-SA 3.0 IGO. n.d.

[7] Costing the National Strategic Plan on Prevention and Control of Cervical Cancer. 2020.

[8] Gallagher K.E., LaMontagne D.S., Watson-Jones D. (2018). Status of HPV vaccine introduction and barriers to country uptake. Vaccine.

[9] Tsedenbal B., Yoshida T., Enkhbat B., Gotov U., Sharkhuu E., Saio M. (2018). Human papillomavirus genotyping among women with cervical abnormalities in Ulaanbaatar. Mongolia Int J Infect Dis.

[10] Pan American Health Organization. PROVAC Toolkit; n.d. Available from: https://www.paho.org/provac-toolkit/tools/about-univac/.

[11] Lusvan M.-E., Debellut F., Clark A., Demberelsuren S., Otgonbayar D., Batjargal T. (2019). Projected impact, cost-effectiveness, and budget implications of rotavirus vaccination in Mongolia. Vaccine.

[12] Anwari P., Debellut F., Vodicka E., Clark A., Farewar F., Zhwak Z.A. (2020). Potential health impact and cost-effectiveness of bivalent human papillomavirus (HPV) vaccination in Afghanistan. Vaccine.

[13] Vodicka E., Nonvignon, Antwi-Agyei K.O., Bawa J., Clark A., Pecenka C., LaMontagne D.S. (2021). The projected cost-effectiveness of HPV vaccine introduction in Ghana. Vaccine.

[14] Vaccine Pricing: Gavi Transitioning Countries. World Health Organization; 2017. Available online at: https://lnct.global/wp-content/uploads/2018/02/Vaccine-Pricing-for-GAVI-Transitioning-Countries-1.pdf.

[15] Vaccine Product, Price and Procurement (V3P). World Health Organization. MI4A: Vaccine Purchase Data; n.d. Available at: https://www.who.int/immunization/programmes_systems/procurement/v3p/platform/module1/en/. Accessed online 4/30/2019.

[16] Salomon J.A., Haagsma J.A., Davis A., de Noordhout C.M., Polinder S., Havelaar A.H. (2015). Disability weights for the Global Burden of Disease 2013 study. Lancet Glob Heal.

[17] Beck J.R., Pauker S.G., Gottlieb J.E., Klein K., Kassirer J.P. (1982). A convenient approximation of life expectancy (the “DEALE”). II. Use in medical decision-making. Am J Med.

[18] Sundaram N., Chen C., Yoong J., Luvsan M.-E., Fox K., Sarankhuu A. (2017). Cost-effectiveness of 13-valent pneumococcal conjugate vaccination in Mongolia. Vaccine.

[19] Woods B., Revill P., Sculpher M., Claxton K. (2016). Country-Level Cost-Effectiveness Thresholds: Initial Estimates and the Need for Further Research. Value Heal.

[20] Spinner C., Ding L., Bernstein D.I., Brown D.R., Franco E.L., Covert C. (2019). Human papillomavirus vaccine effectiveness and herd protection in young women. Pediatrics.

[21] Tabrizi S.N., Brotherton J.M.L., Kaldor J.M., Skinner S.R., Liu B., Bateson D. (2014). Assessment of herd immunity and cross-protection after a human papillomavirus vaccination programme in Australia: A repeat cross-sectional study. Lancet Infect Dis.

[22] Chow E.P.F., Machalek D.A., Tabrizi S.N., Danielewski J.A., Fehler G., Bradshaw C.S. (2017). Quadrivalent vaccine-targeted human papillomavirus genotypes in heterosexual men after the Australian female human papillomavirus vaccination programme: a retrospective observational study. Lancet Infect Dis.

[23] Kim J.J., Sharma M., O'Shea M., Sweet S., Diaz M., Sancho-Garnier H. (2013). Model-based impact and cost-effectiveness of cervical cancer prevention in the extended middle east and North Africa (EMENA). Vaccine.

[24] Kim J.J., Campos N.G., O’Shea M., Diaz M., Mutyaba I. (2013). Model-based impact and cost-effectiveness of cervical cancer prevention in sub-Saharan Africa. Vaccine.

[25] Okeah B.O., Ridyard C.H. (2020). Factors Influencing the Cost-Effectiveness Outcomes of HPV Vaccination and Screening Interventions in Low-to-Middle-Income Countries (LMICs): A Systematic Review. Appl Health Econ Health Policy.

