# Supplementary material for: The potential cost-effectiveness of HPV vaccination among girls in Mongolia
Source: Vaccine X. 2022 Apr 8;11:100161. doi: 10.1016/j.jvacx.2022.100161 (PMC9059071; doi:10.1016/j.jvacx.2022.100161)
Supplement: Supplementary data 1 [file mmc1.docx]

**Supplementary Appendix**

*Supplementary Appendix Table 1. Vaccine Efficacy (Targeted Protection + Cross-Protection)*

| **High Risk HPV Types** | **Quadrivalent (Gardasil^®^) Vaccine Protection** | **Quadrivalent (Gardasil^®^)**  **Vaccine Efficacy** | **Bivalent (Cervarix^TM^) Vaccine Protection** | **Bivalent (Cervarix^TM^)**  **Vaccine Efficacy** | **Estimated type % among all cancer cases in Mongolia** [9] |
| --- | --- | --- | --- | --- | --- |
| 16/18 | Targeted Protection | Girls: 94.3% (88.1-99.6%) [15] | Targeted Protection | Girls: 94.3% (66.6, 99.1%) [30] | 64.3% |
| 31 | Low Cross-Protection | Girls: 70.0% (32.1-88.2%) [5] | High Cross-Protection | Girls: 89.4% (65.5-97.9%) [5] | 7.1% |
| 33 | Non-significant | N/A | High Cross-Protection | Girls: 82.3% (53.4-94.7%) [5] | 14.3% |
| 45 | Non-significant | N/A | High Cross-Protection | Girls: 100% (41.7-100%) [5] | Not identified |
